# Neuropilin-1 expression modulates infection susceptibility to murine cytomegalovirus at the materno-fetal interface

**DOI:** 10.1128/jvi.01610-25

**Published:** 2025-11-25

**Authors:** Luís Fonseca Brito, Eléonore Ostermann, Julian Kottlau, Silvia Tödter, Renke Brixel, Roland Schüle, Maria Emilia Solano, Wolfram Brune, Felix Rolf Stahl

**Affiliations:** 1 Institute of Immunology, University Medical Center Hamburg-Eppendorf 37734, Hamburg, Germany; 2 Leibniz Institute of Virology 28367, Hamburg, Germany; 3 Center for Clinical Research, University of Freiburg Medical Center, Freiburg, Germany; 4 Laboratory of Translational Perinatology, Department of Obstetrics and Gynecology, University of Regensburg 9147, Regensburg, Germany; University of Virginia, Charlottesville, Virginia, USA

**Keywords:** CMV, congenital, cytomegalovirus, interferon, MCMV, neuropilin-1, NRP1, placenta, trophoblast

## Abstract

Human cytomegalovirus (HCMV) is the leading infectious cause of congenital disease, but the mechanisms governing vertical transmission remain poorly defined. Murine cytomegalovirus (MCMV) infection in mice is a widely used model to study pathogenesis *in vivo*. However, the mouse model cannot be used to study congenital infection as viral transmission from mother to fetus *in utero* does not occur. In this study, we investigated tissue-specific features of the murine materno-fetal barrier that may restrict viral infection. Following high-dose intravenous challenge of wild-type and severely immunocompromised pregnant mice, MCMV replicated efficiently in the maternal liver but infected only a few cells in the placenta, suggesting an intrinsic resistance. *Ex vivo* analyses of primary placental cells, trophoblast stem cells, and a trophoblast cell line revealed a low susceptibility to MCMV infection compared to other permissive cell types. The resistance of trophoblast cells to MCMV infection correlated with the absence of neuropilin-1 (NRP1), a cellular receptor required for efficient infection of several cell types. Enforced expression of NRP1 in trophoblast cells increased their susceptibility to MCMV infection and replication, suggesting that the resistance of trophoblast cells to MCMV infection is caused by the lack of a critical receptor. These findings further suggest that, in addition to immune-mediated restrictions, cell-intrinsic resistance limits MCMV infection and transmission at the murine materno-fetal interface.

**IMPORTANCE:**

Congenital cytomegalovirus (CMV) infection is a major cause of developmental disabilities in newborns, yet the biological factors that influence transmission from mother to fetus remain unclear. In this study, we demonstrate that trophoblast cells of the murine placenta are naturally resistant to CMV infection as they lack expression of a host protein, neuropilin-1 (NRP1), that the virus requires for entry. By introducing this protein into resistant cells, we demonstrated that susceptibility to infection can be reinstated, indicating that the absence of NRP1 plays a key protective role at the materno-fetal barrier. These results provide insight into why mice rarely transmit CMV to their offspring and how species-specific differences in placental biology shape susceptibility. Understanding these mechanisms will aid in refining animal models and may help identify new targets to prevent congenital infection in humans.

## INTRODUCTION

Human cytomegalovirus (HCMV) is a large dsDNA virus and a member of the *Betaherpesvirinae* subfamily. It is highly prevalent in the human population and generally regarded as an opportunistic pathogen. During pregnancy, vertical transmission of the virus from the mother to the fetus may cause significant clinical symptoms, and HCMV is the leading pathogen responsible for permanent disabilities in neonates (1). Determinants responsible for the high variation in vertical transmission events of HCMV-infected pregnant women and origin of clinical symptoms are currently under investigation and likely include both host defense mechanisms and viral pathogenicity factors.

Several animal models are applied to study cytomegalovirus (CMV)–host interaction. The primary advantage of the rhesus macaque CMV and guinea pig CMV models for studying CMV pathogenesis lies in their capacity to replicate fetal infection within their respective species (2–4). Nevertheless, the use of these models is restricted to a limited number of research centers, primarily due to ethical restraints, the complexity of animal husbandry, and the limited availability of species-specific tools. As a result, murine cytomegalovirus (MCMV) infection in mice remains the most widely used *in vivo* model for CMV research. However, vertical transmission *in utero* does not occur in the commonly used inbred mice and laboratory MCMV strains (5–9). Infection of fetal membranes has been observed in severe combined immunodeficient (SCID) mice (10) and the offspring of latently infected mice (11). Therefore, current models for *in utero* MCMV infection involve invasive procedures such as direct injection of virus into the placenta or the fetal brain (12–14). Alternatively, infection of neonatal mice with MCMV is a well-established surrogate model for congenital CMV disease (12, 13, 15).

The materno-fetal interface is the site defined by the implantation and invasion of the blastocyst-derived placenta into the uterine stroma, the decidua. The placenta serves not only as a conduit for nutrients and waste but also as a critical immunological barrier that protects the developing fetus from maternal pathogens (16). Although the structural and organizational features of the human and mouse placenta differ, each species has evolved to maintain an optimized immunological equilibrium essential for sustaining a healthy pregnancy (17). Thus, mechanisms of placental resistance—structural, cellular, and immunological—are likely involved in limiting vertical transmission of MCMV. A range of host molecules, including epidermal growth factor receptor, integrin αvβ3, CD90, CD147, neuropilin-2 (NRP2), and platelet-derived growth factor receptor alpha (PDGFRα), have been characterized as receptors or co-receptors mediating HCMV entry into fibroblasts, epithelial cells, and endothelial cells (18–23). Less is known about host receptors relevant to MCMV infection. NRP1 and the major histocompatibility complex I (MHC-I) were described as crucial host factors for MCMV infection of endothelial cells and macrophages, respectively (24, 25). The roles of other putative receptors, such as Integrin β1 subunit (ITGβ1) and PDGFRα, remain to be defined (26, 27).

Here, we addressed tissue-specific features of the murine materno-fetal barrier that could play a role in interfering with vertical MCMV transmission. Infection events were tracked in the materno-fetal barrier lining cells, including trophoblast cells, *in vivo* and *ex vivo*. A trophoblast cell line and murine trophoblast stem cells (mTSCs) were used to identify tissue-specific host factors for MCMV replication. The results of this study suggest that a combination of both host immunity and intrinsic properties of tissue cells contributes to MCMV resistance in the materno-fetal interface.

## RESULTS

### Tissue-specific MCMV replication in placenta and maternal liver under conditions of immune suppression

Lymphocytes, such as T and natural killer (NK) cells, as well as type I interferons, are well established to interfere with MCMV infection. *Rag2^-/-^Il2rg^-/-^* mice are deficient in functional B, T, and NK cells, while *Ifnar1^-/-^* mice lack a crucial subunit of the type-I IFN receptor, resulting in defective interferon signaling. These models of immunodeficiency exhibit augmented MCMV replication in *Rag2^-/-^Il2rg^-/-^* (28, 29) or *Ifnar1^-/-^* (30) mice. To address whether impaired immunity impacts viral replication in placental tissue, *Rag2^-/-^Il2rg^-/-^* and *Ifnar1^-/-^* or wild-type (WT) pregnant mice were infected intravenously (i.v.) with MCMV-3DR, here referred to as MCMV, which is a recombinant expressing mCherry and *Gaussia* luciferase (see Materials and Methods). The biological fitness of this virus has been demonstrated in several *in vivo* studies (24, 28, 31, 32) and is used throughout the present investigation. The dams were sacrificed on 3–9 days post-infection (dpi) to prevent a reduced health status normally occurring in *Rag2^-/-^Il2rg^-/-^* and *Ifnar1^-/-^* pregnant mice upon infection with high MCMV doses. The average observation time after infection was 5.3 days (Table 1). Maternal livers and conceptuses (embryo/fetus, placenta, and associated membranes) were investigated to monitor infection events in tissue by means of mCherry expression in single cells.

**TABLE 1:** Overview of MCMV exposure and detection in conceptuses^*a*^

Genotype	WT		*Rag2* ^-/-^ *Il2rg* ^-/-^		*Ifnar1* ^-/-^	
Dams	4	(#1, #2, #3, #4)	5	(#1, #2, #3, #4, #5)	3	(#1, #2, #3)
Gestational day	9-12	(9, 10, 12, 12)	11–14	(11, 11, 14, 14, 14)	10–12	(10, 12, 12)
Days post-infection	4-7	(7, 6, 4, 4)	3–9	(7, 9, 3, 3, 5)	5–6	(6, 5, 5)
Conceptus analyzed	31	(8, 10, 8, 5)	25	(3, 5, 9, 3, 5)	22	(7, 5, 10)
Sections analyzed	203	(64, 80, 31, 28)	148	(21, 29, 36, 26, 62)	98	(46, 20, 32)
MCMV^+^ sections	0 (0 %)	(0, 0, 0, 0)	11 (7.4 **%**)	(0, 8, 1, 0, 2)	0 (0 **%**)	(0, 0, 0)

^*a*^ This table provides the time of MCMV application during pregnancy (gestational day), the time of analysis post-infection (days post-infection), the number of conceptuses analyzed per dam, the number of histology sections analyzed per dam, and the number of sections where MCMV-infected cells were detected. Values in parentheses correspond to data obtained from individual dams.

**Fig 1:**
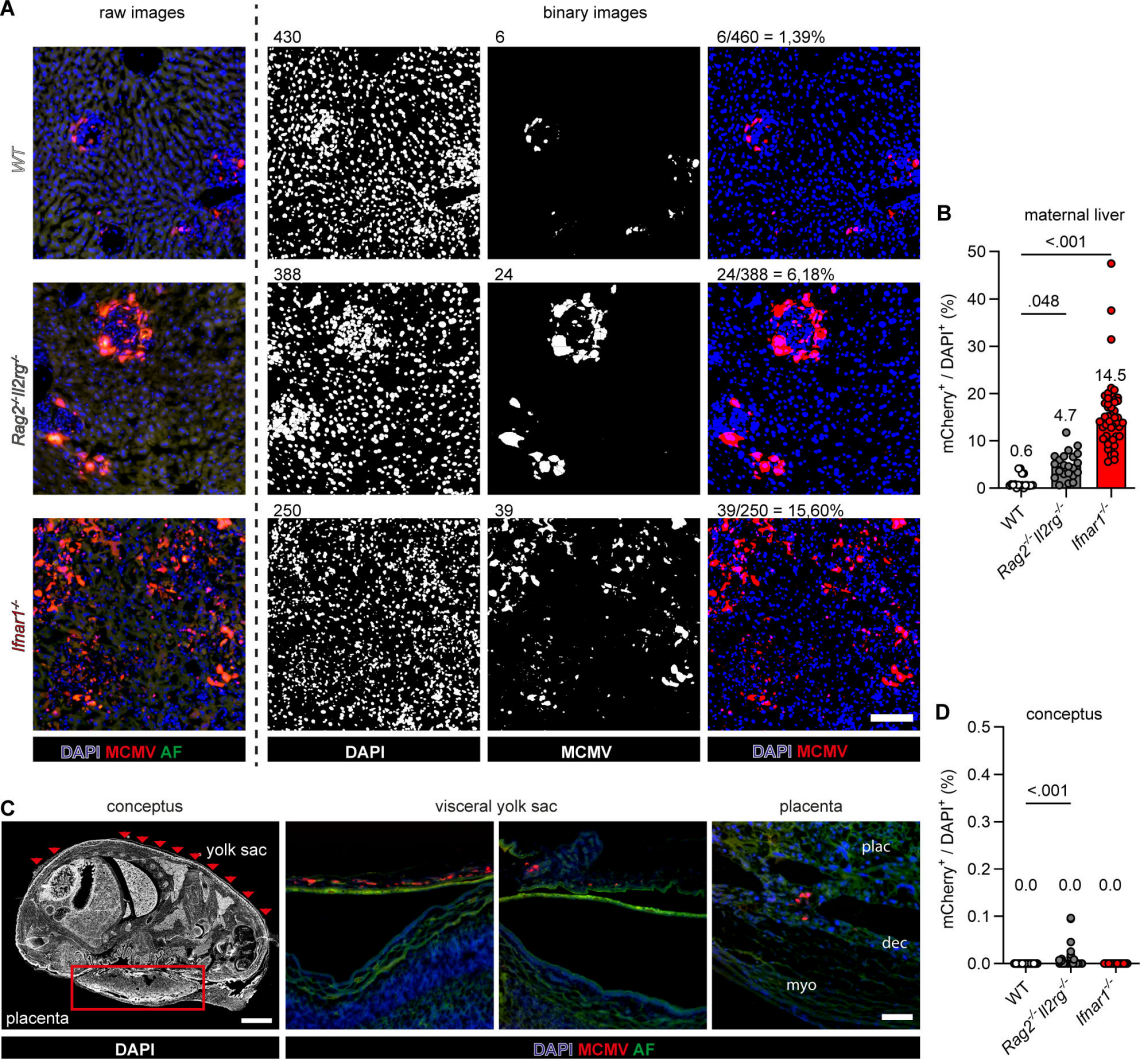
Differential MCMV susceptibility of placenta and liver. *WT*, *Rag2*^-/-^*Il2rg*^-/-^, or *Ifnar1^-/-^* dam was intravenously infected with MCMV (1 × 10^6^ PFU/dam) and livers as well as conceptuses were analyzed 5 (for *Rag2^-/-^Il2rg^-/-^* mice) or 6 (for *WT* and *Ifnar1^-/-^* mice) days post-infection (dpi) by fluorescence microscopy for the number of mCherry^+^ MCMV-infected cells. (**A**) Microscopy images of maternal livers illustrate MCMV-infected (mCherry^+^) cells (red) and nuclear staining (blue) before (raw images) and after data processing (binary images). Numbers indicate automated counting events of fluorescence signals for nuclei (DAPI), mCherry^+^ cells (MCMV), and the mCherry / DAPI ratio. Scale 100 µm. (**B**) Quantification of MCMV-infected cells per parenchyma cells of maternal livers. The data were pooled from various dpi (from Table 1), and medians are depicted. (**C**) Representative image of a conceptus as acquired for data analysis. Red arrows and rectangles indicate the position of observed areas of infection in the visceral yolk sac and placenta of a *Rag2^-/-^Il2rg^-/-^* conceptus. MCMV-infected cells are shown in red, and autofluorescence (AF) of the tissue in green. Plac: placenta; dec: decidual stroma; myo: myometrium. Scales 2,000 µm (left for conceptus) and 100 µm (right for visceral yolk sac and placenta). (**D**) Quantification of MCMV-infected cells per conceptus applying the same data processing setup as in (**A**). The data were pooled from various dpi (from Table 1), and medians are depicted. (B + D) *P*-values were calculated using the Kruskal-Wallis nonparametric unpaired test and Dunn’s multiple comparisons test.

This method holds the advantage of minimizing false-positive results, which are more likely to occur with nucleic acid amplification techniques or luciferase activity assays due to contamination with maternal blood. Expression of MCMV-encoded mCherry and *Gaussia* luciferase correlates with virus titers *in vitro* (31) and *in vivo* (28). Histology sections of maternal livers were used to establish a standardized algorithm (see Materials and Methods) to analyze the frequency of mCherry^+^ MCMV-infected cells per DAPI^+^ nuclei to estimate the magnitude of virus load in tissue (Fig. 1A). Both *Rag2^-/-^Il2rg^-/-^* and *Ifnar1^-/-^* mice exhibited more infected cells in the liver as compared to WT mice, confirming that reduced host immunity allowed increased virus replication in pregnant mice (Fig. 1B). In contrast, histological analysis of conceptuses revealed low numbers of MCMV-infected cells in the visceral yolk sac and placenta (Fig. 1C and D). These data suggested that, unlike the liver, the placenta exhibited biological resistance to MCMV infection.

### Placental and decidual cells exhibit intrinsic resistance to MCMV infection *ex vivo*

**Fig 2:**
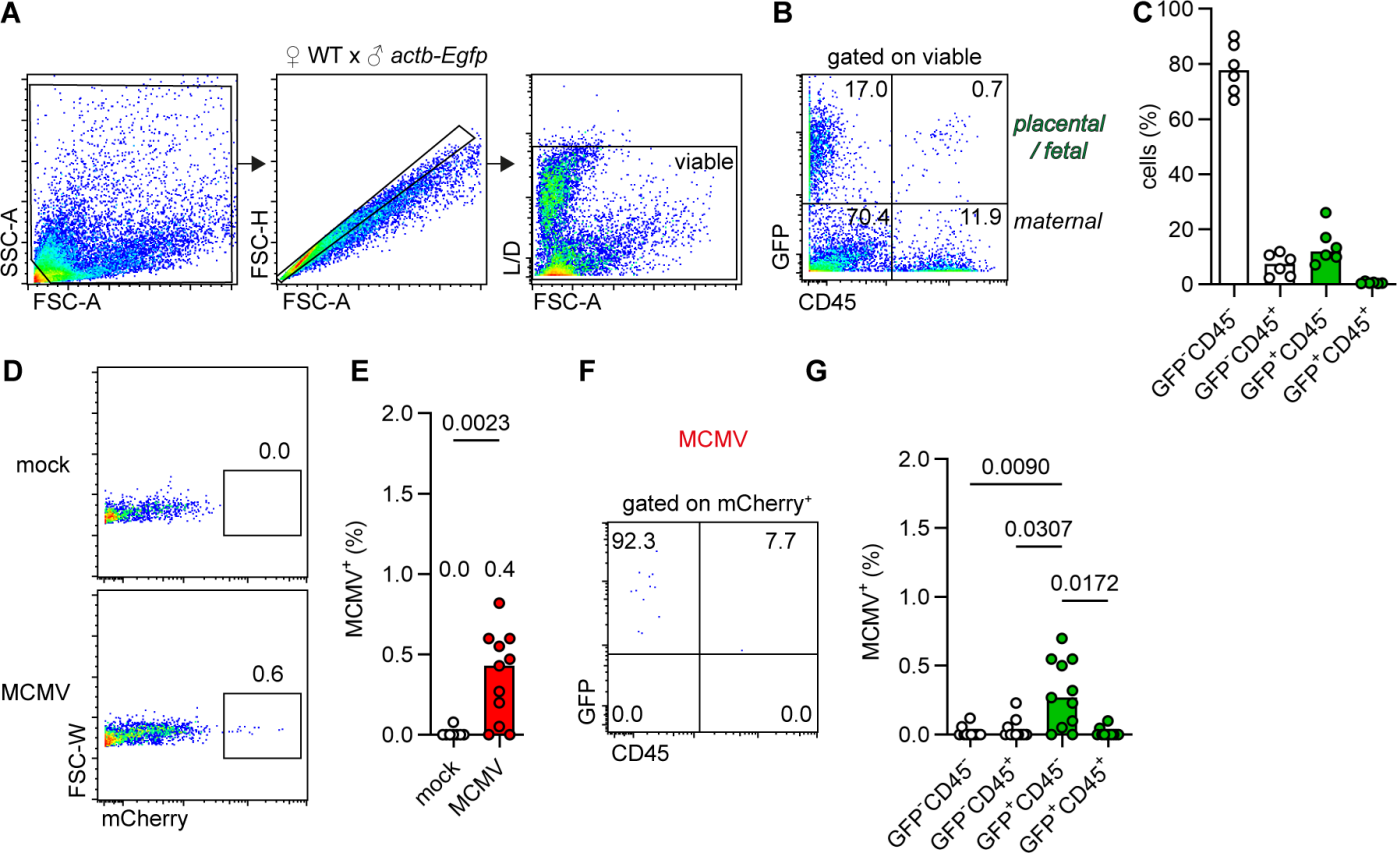
Placental and decidual cells exhibit intrinsic resistance to MCMV infection *ex vivo.* Primary murine materno-fetal barrier lining cells were isolated and analyzed for their susceptibility to MCMV infection. (**A**) Gating strategy for (**B–-G**) flow cytometry analysis of primary cells obtained from decidua-lined placentas from *WT* dams mated with *actb-Egfp* male mice. L/D: live/dead staining. (**B**) Flow cytometry dot plots of cells (**A**) directly after isolation. GFP^+^ cells are cells of placental/fetal origin, and CD45^+^ cells are of hematopoietic origin. (**C**) Percentage of the indicated cell subpopulations as in (**B**). White depicts cells of maternal and green cells of placental/fetal origin. Data shown are medians from *n* = 6 placenta preparations of *n* = 2 dams. (**D–G**) Primary cells were infected directly after isolation with MCMV (MOI 0.5). (**D**) Flow cytometry dot plots and (**E**) percentage of MCMV-infected (mCherry^+^) among viable cells after 1 dpi are shown. (**F**) Flow cytometry dot plot gated on MCMV-infected (mCherry^+^) subpopulation, and (**G**) frequency of MCMV-infected among the four different subpopulations identified by GFP or CD45 expression is shown. Representative dot plots and medians of two independent experiments with *n* = 12 pooled placenta preparations of *n* = 4 dams are depicted. Significance was calculated with the parametric paired one-way ANOVA with Tukey’s multiple comparisons test.

The materno-fetal interface consists of the blastocyst-derived placenta and the decidua. The placenta is composed of successive layers of trophoblast cells, followed by fetal endothelial capillaries that separate the maternal from the fetal blood circulation (33). To assess the susceptibility of materno-fetal interface-lining cells to MCMV infection, placental and decidual cells were obtained from the progeny of WT dams mated to homozygous *actb-Egfp* male mice. The offspring and placentas generated by this mating strategy are heterozygous for *actb-Egfp*, exhibit ubiquitous GFP expression, and thus allow for distinction between maternal decidual stroma and placenta cells. Cell suspensions obtained from the decidua-lined placenta were analyzed for GFP and CD45 expression to classify cells as fetal/placental (GFP^+^) or maternal (GFP^−^) and hematopoietic (CD45^+^) or non-hematopoietic (CD45^−^) cells (Fig. 2A and B). As the placenta invades the maternal decidua at the implantation site, in these tissue preparations, cells of maternal origin outnumbered those of fetal origin, including both hematopoietic and non-hematopoietic cells (Fig. 2C). There was a distinct CD45^−^ placental cell population but only very few fetal hematopoietic cells (Fig. 2C). These primary cell suspensions were infected with MCMV directly after isolation and analyzed 1 day post-infection for MCMV-encoded mCherry expression. A small fraction of the cultured cells was susceptible to MCMV infection (Fig. 2D and E). MCMV-infected cells almost exclusively belonged to the placental non-hematopoietic cell population, whereas very few maternal decidual cells were mCherry^+^ (Fig. 2F and G). Hence, *ex vivo* decidua and placenta cells exhibited a significant resistance to MCMV as observed *in vivo* in *WT* and immunocompromised animals.

### Placental SM9-1 cells display marked resistance to MCMV compared with diverse cell lines

**Fig 3:**
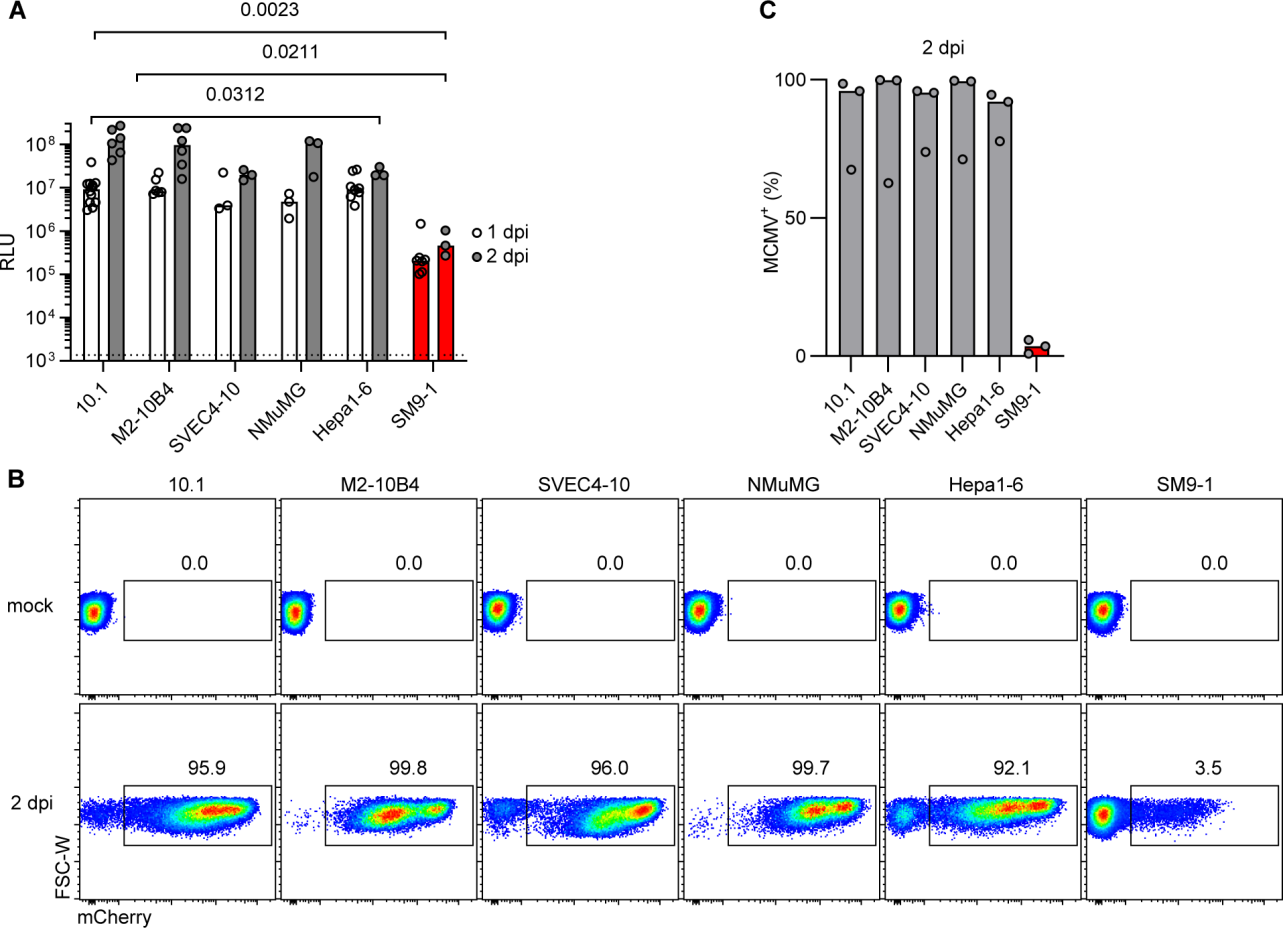
SM9-1 trophoblast cells are resistant to MCMV infection. Cell lines of diverse tissue origins were comparatively infected with MCMV (MOI of 0.5), and (**A**) luciferase activity in cell culture supernatants was measured 1 or 2 dpi as indicated. The dotted line indicates the detection limit determined by the mean background luciferase activity of uninfected 10.1 cells. Medians of *n* ≥ 3 independent experiments are shown. *P*-values were calculated using a two-way ANOVA with Šídák’s multiple comparisons test. (**B**) Flow cytometry dot plots of MCMV-encoded mCherry expression in uninfected (mock) cells of different lines and at 2 dpi. mCherry^+^ cells are MCMV-infected. Representative dot plots of *n* ≥ 3 independent experiments are depicted. (**C**) Percentage of mCherry^+^ MCMV-infected cells at 2 dpi. Medians of *n* = 3 independent experiments are shown.

To assess the relative susceptibility of placental cells to MCMV infection, the SM9-1 placental trophoblast cell line (34) was comparatively analyzed alongside a panel of cell lines derived from diverse mouse tissues. Cell lines that were described to be permissive for MCMV or even used to propagate the virus to generate virus stocks, such as the 10.1 murine embryo fibroblasts (35), M2-10B4 bone marrow stromal cells (36), SVEC4-10 lymphatic endothelial cells (37), and NMuMG mammary gland epithelial cells (38), were included in the analysis. In addition, the Hepa1-6 liver cell line (39) was added as MCMV infection of the liver was in distinct contrast to placental tissue *in vivo* (Fig. 1). High and robust expression of MCMV-encoded *Gaussia* luciferase was found in cell culture supernatants of all but the SM9-1 cell lines after 1 and 2 days of MCMV infection (Fig. 3A). On average, MCMV infection of known permissive cells led to a 40-fold and a 141-fold higher luciferase activity than infection of SM9-1 cells at 24 h and 48 h post-infection, respectively. Similarly, high percentages of mCherry^+^ cells were detected in all cell types except for SM9-1 cells at 2 dpi (Fig. 3B). Only a small fraction of SM9-1 cells expressed the MCMV-encoded fluorophore (Fig. 3C). These data indicated that SM9-1 trophoblast cells are poorly infectible and therefore relatively resistant to MCMV infection.

### Absence of NRP1 in SM9-1 trophoblast cells correlates with resistance to MCMV infection

**Fig 4:**
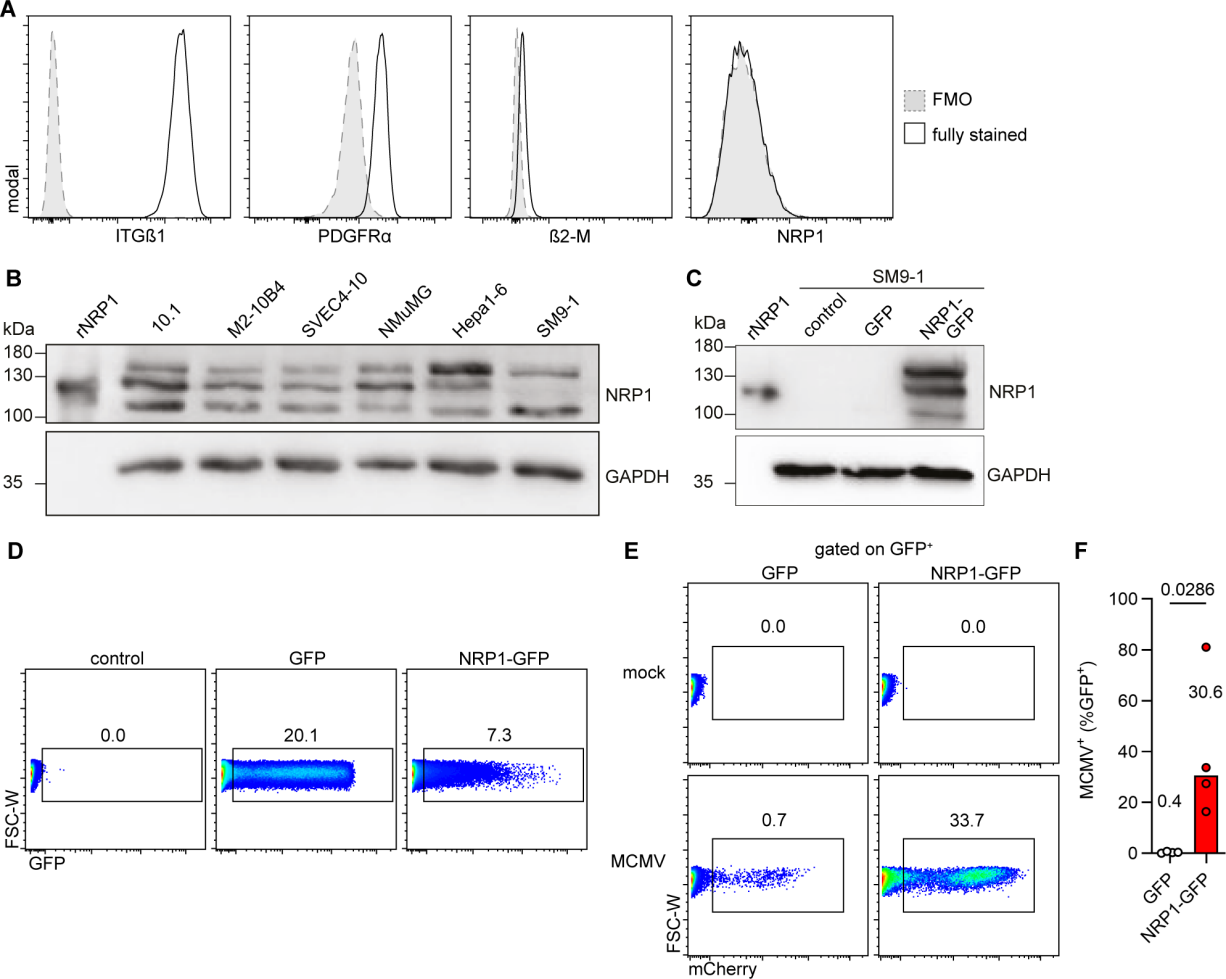
Absence of NRP1 in SM9-1 trophoblast cells correlates with resistance to MCMV infection. SM9-1 cells were characterized for expression of MCMV host factors, transfected with NRP1-encoding plasmids, and analyzed for MCMV susceptibility. (**A**) Flow cytometry histograms of SM9-1 cells stained for putative entry receptors as indicated. FMO: fluorescence minus one. Representative data of *n* ≥ 3 experiments are depicted. (**B**) Cell lysates of different cell lines were harvested and analyzed by immunoblot. Recombinant NRP1 (rNRP1) served as a positive control and GAPDH as a loading control. Representative blots from *n* ≥ 2 experiments are shown. (**C**) SM9-1 cells were left untreated (control) or transfected with GFP or NRP1-GFP-encoding plasmids. Cell lysates were harvested at 2 days post-transfection and analyzed by immunoblot. rNRP1 serves as a positive control, and GAPDH as a loading control. Representative blots from *n* ≥ 2 experiments are shown. (**D**) Flow cytometry dot plots of GFP expression by SM9-1 cells transfected with plasmid-encoding GFP or NRP1-GFP at 2 days after transfection. Transfected cells were mock infected or infected with MCMV (MOI 0.5) and analyzed at 1 dpi. (**E**) Flow cytometry dot plots of MCMV-encoded mCherry expression among the transfected cells (GFP^+^) 1 dpi are shown. (**F**) The percentage of MCMV-infected (mCherry^+^) among the transfected cells (GFP^+^) at 1 dpi is shown. For (**D–F**) representative dot plots and medians of four independent experiments are provided. *P*-values were calculated using the Mann-Whitney U test.

MCMV infection is established through successful viral entry into host cells and ultimately leads to the production of infectious progeny. To understand potential mechanisms of resistance to MCMV, SM9-1 trophoblast cells were analyzed for surface expression of the putative MCMV entry receptors ITGβ1, PDGFRα, β2-microglobulin (β2-M) as part of the MHC-I, and NRP1. SM9-1 trophoblast cells exhibited membrane expression of ITGβ1 and PDGFRα, whereas β2-M was barely detectable, and NRP1 was not expressed (Fig. 4A). NRP1 expression could be confirmed in MCMV-susceptible cell lines 10.1, M2-10B4, SVEC4-10, NMuMG, and Hepa1-6 cells, but not in SM9-1 trophoblast cells (Fig. 4B).

NRP1 is a transmembrane glycoprotein involved in the nervous and vascular system during embryogenesis (40) and was identified as a critical host factor for MCMV infection (25).

In a reanalysis of a recently reported single-nucleus RNA sequencing data set of gestational day 12.5 murine placental cells (41), *Itgb1* was ubiquitously expressed in all cell types. *Pdgfra* and some *B2m* expression were found in decidual stroma cells and trophoblasts. In contrast, *Nrp1* was found to be expressed only in fetal mesenchymal and endothelial cells but virtually absent in decidual stroma and placental trophoblast cells (Fig. S1). Hypothesizing that the lack of NRP1 expression in SM9-1 cells might be responsible for the resistance to MCMV infection, the cells were transfected with plasmids expressing GFP or NRP1-GFP and subsequently infected with MCMV. NRP1 protein was detected in cells transfected with NRP1-GFP but not in cells transfected with GFP or untransfected SM9-1 cells (Fig. 4C). SM9-1 cells transfected with NRP1-GFP were more susceptible to MCMV infection than cells transfected with GFP (Fig. 4D and E), leading to a substantially higher percentage of mCherry^+^ cells at 1 dpi (Fig. 4F). These results suggested that the resistance of SM9-1 trophoblast cells to MCMV infection may be due to a lack of NRP1 expression.

### NRP1 expression overcomes the resistance of SM9-1 trophoblast cells to MCMV

**Fig 5:**
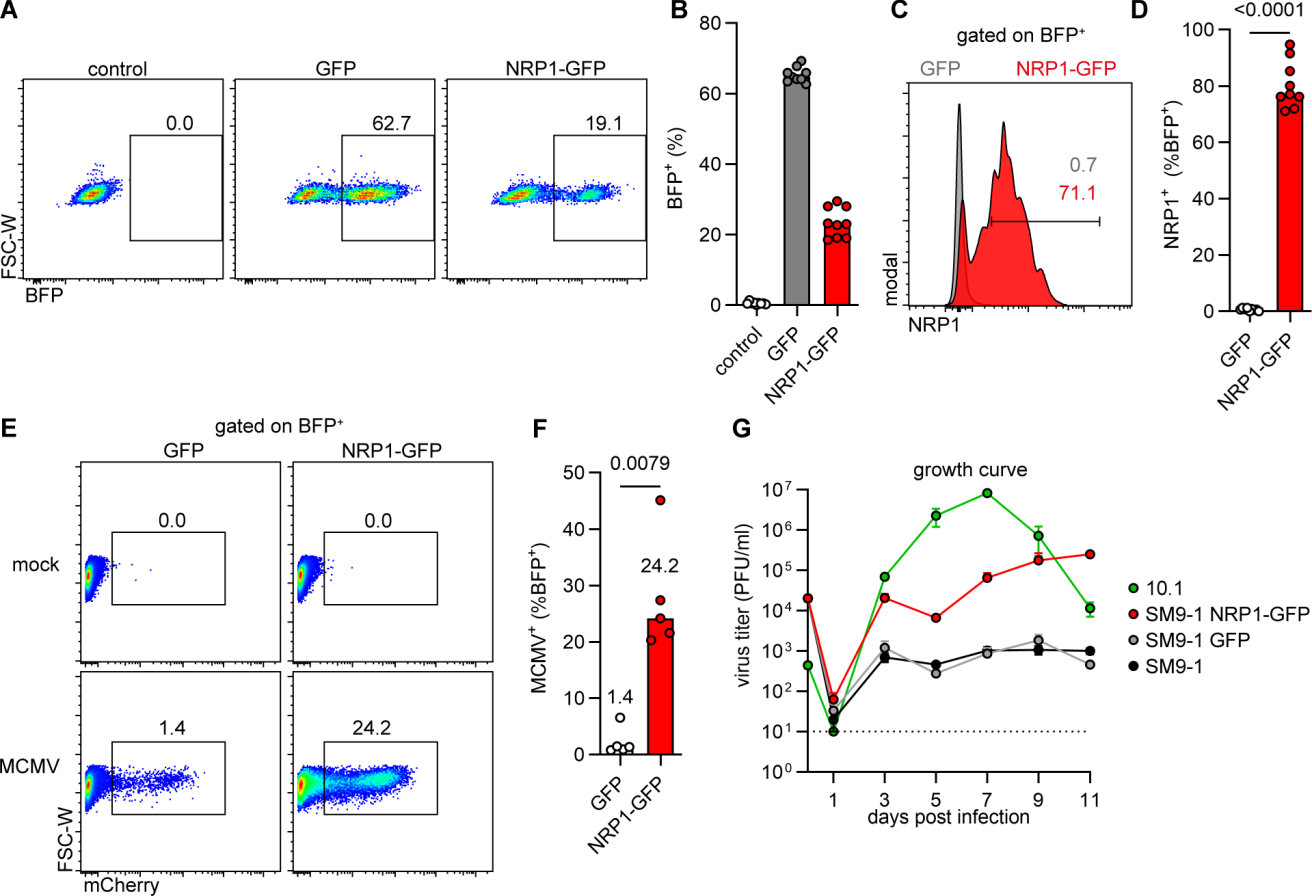
MCMV infection of SM9-1 trophoblast cells transduced with NRP1. Stable expression of GFP or NRP1-GFP was introduced in SM9-1 trophoblast cells using lentiviral vectors with subsequent analysis for MCMV susceptibility. (**A–D**) SM9-1 cells were transduced with lentiviral vectors expressing GFP or NRP1-GFP and BFP-puro as a selectable marker. (**A**) Flow cytometry dot plots and (**B**) quantification of transduction efficacy (BFP expression) of GFP and NRP1-GFP-encoding constructs. (**C**) Flow cytometry histogram and (**D**) quantification of NRP1 expression on transduced cells as indicated. Representative flow cytometry images and medians of *n* = 9 independent experiments are shown. *P*-values were calculated using the Mann-Whitney U test. (**E and F**) Transduced cells, as in (**A–D**), were infected with MCMV (MOI 0.5) and analyzed at 1 dpi. (**E**) Flow cytometry dot plots and (**F**) quantification of MCMV-infected (mCherry^+^) cells gated on the transduced (BFP^+^) cells. Representative dot plots and medians of *n* = 5 independent experiments are shown. *P*-values were calculated using the Mann-Whitney U test. (**G**) Multistep replication kinetics of MCMV on 10.1 fibroblasts (infected at MOI 0.1) and SM9-1 cells transduced and not transduced as indicated (infected at an MOI of 1). The virus released into the supernatant was titrated on M2-10B4 cells. Mean ± SEM of triplicates is shown. The dotted line indicates the detection limit.

As plasmid transfection allowed vigorous but only transient overexpression of NRP1 (Fig. 3), the impact of stable NRP1 expression on the infectability of SM9-1 trophoblast cells was tested. To do this, NRP1-GFP and GFP coding sequences were inserted into a lentiviral vector that expresses BFP and a puromycin resistance marker. SM9-1 cells were transduced with these lentiviral vectors and selected with puromycin. Using the NRP1-GFP-encoding construct for transduction led to a lower frequency of BFP-expressing cells than the GFP-encoding control (Fig. 5A and B). NRP1 cell surface expression was detected by flow cytometry in cells transduced with NRP1-GFP but not in cells transduced with GFP (Fig. 5C and D). SM9-1 cells transduced with NRP1-GFP were more susceptible to MCMV infection than cells transduced with GFP (Fig. 5E), leading to a 17-fold increase in mCherry^+^ cells at 1 dpi (Fig. 5F). To test whether NRP1-expressing SM9-1 cells are permissive for MCMV replication, we used transduced and non-transduced SM9-1 cells and 10.1 fibroblasts for multistep replication kinetics. As expected, 10.1 fibroblasts were highly permissive and produced high titers of viral progeny (Fig. 5H). By contrast, SM9-1 cells transduced with GFP or non-transduced produced relatively low MCMV titers. However, NRP1-GFP-expressing SM9-1 cells produced up to 100-fold higher titers (Fig. 5H). Together, these results demonstrated that the resistance of SM9-1 trophoblast cells to MCMV infection could be overcome by NRP1 expression.

### Murine trophoblast stem cells lack NRP1 expression and are resistant to MCMV infection

**Fig 6:**
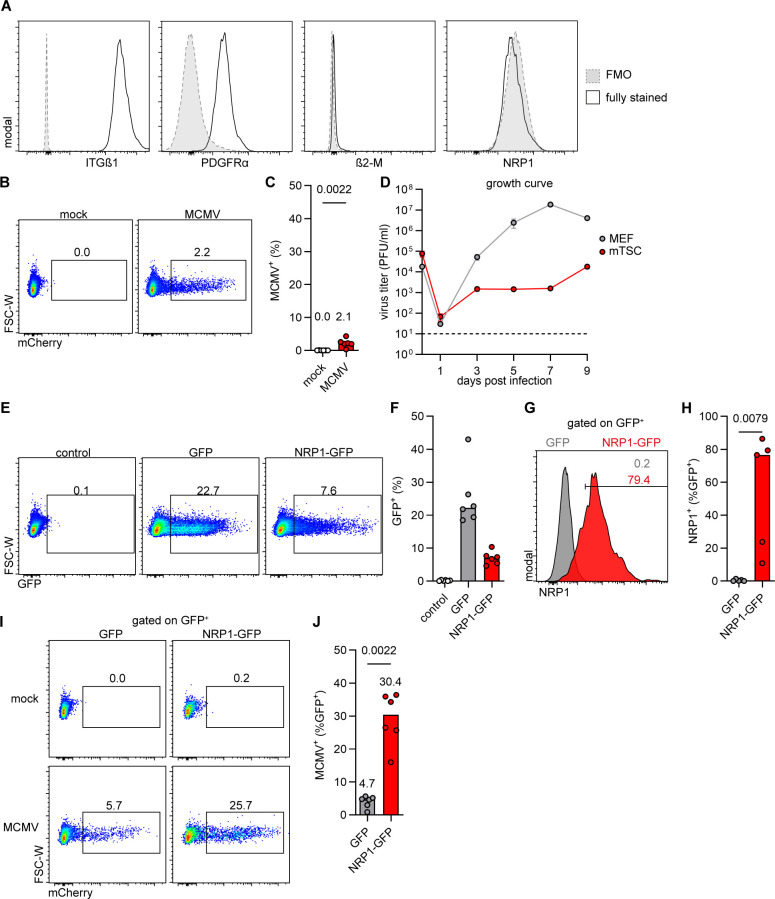
Absence of NRP1 expression in mTSCs and resistance to MCMV infection. mTSCs were characterized for the expression of MCMV host factors, transfected with NRP1-encoding plasmids, and analyzed for MCMV susceptibility. (**A**) Flow cytometry histograms of mTSCs stained for the receptors as indicated. FMO: fluorescence minus one. Representative data of *n* = 3 independent experiments is depicted. (**B and C**) mTSCs were infected with MCMV (MOI 0.5) and analyzed at 1 dpi. (**B**) Flow cytometry dot plots and (**C**) quantification of MCMV-infected (mCherry^+^) mTSCs. Representative flow cytometry images and medians of *n* = 6 independent experiments are shown. *P*-values were calculated using the Mann-Whitney U test. (**D**) Multistep replication kinetics of MCMV on mouse embryonic fibroblasts (MEFs) (MOI 0.1) and mTSCs (MOI 1). The virus released into the supernatant was titrated on M2-10B4 cells. Mean ± SEM of triplicates is shown. The dotted line indicates the detection limit. (**E-H**) mTSCs were transfected with plasmids encoding GFP or NRP1-GFP and analyzed at 1 day post-transfection. (**E**) Flow cytometry dot plots and (**F**) percentage of GFP-expressing cells in the indicated populations. Representative flow cytometry images and medians of *n* = 6 independent experiments are shown. (**G**) Flow cytometry histogram and (**H**) quantification of NRP1 expression among the transfected (GFP^+^) cells. Representative flow cytometry image and medians of *n* = 6 independent experiments are shown. *P*-values were calculated using the Mann-Whitney U test. (**I and J**) mTSCs were transfected with plasmids encoding GFP or NRP1-GFP, infected at 1 day post-transfection with MCMV (MOI 0.5), and analyzed at 1 dpi. (**I**) Flow cytometry dot plots and (**J**) quantification of MCMV-infected (mCherry^+^) among transfected (GFP^+^) cells. Representative flow cytometry images and medians of *n* = 6 independent experiments are shown. *P*-values were calculated using the Mann-Whitney U test.

mTSCs represent cells of the trophoblast lineage and retain the capacity to differentiate *in vitro* (33, 42). In the present study, previously described mTSCs isolated from E3.5 blastocysts were used (43). For quality control, visual confirmation by microscopy was performed together with cellular marker gene expression analysis by quantitative real-time RT-PCR (Fig. S2). Next, cell surface expression of putative MCMV entry receptors in mTSCs was analyzed by flow cytometry. Similarly to the SM9-1 cell line, mTSCs expressed both ITGβ1 and PDGFRα but little β2-M and no NRP1 (Fig. 6A). A small fraction of the mTSCs was susceptible to MCMV infection (Fig. 6B and C), leading to low levels of progeny production in replication kinetics experiments (Fig. 6D).

To test whether enforced NRP1 expression can increase MCMV infection, mTSCs were transfected with plasmids expressing GFP or NRP1-GFP (Fig. 6E and F) and subsequently infected with MCMV. Flow cytometric analysis confirmed NRP1 surface expression in mTSCs transfected with NRP1-GFP but not with GFP (Fig. 6G and H). NRP1-GFP transfection increased MCMV susceptibility in mTSCs, leading to a significantly higher percentage of mCherry^+^ cells at 1 dpi than in control cells (Fig. 6I and J). These findings demonstrate that mTSCs lack NRP1 expression and are resistant to MCMV infection, paralleling the phenotype observed in SM9-1 trophoblasts and primary placental cells.

### Inducible NRP1 expression increases MCMV infection in mTSCs

**Fig 7:**
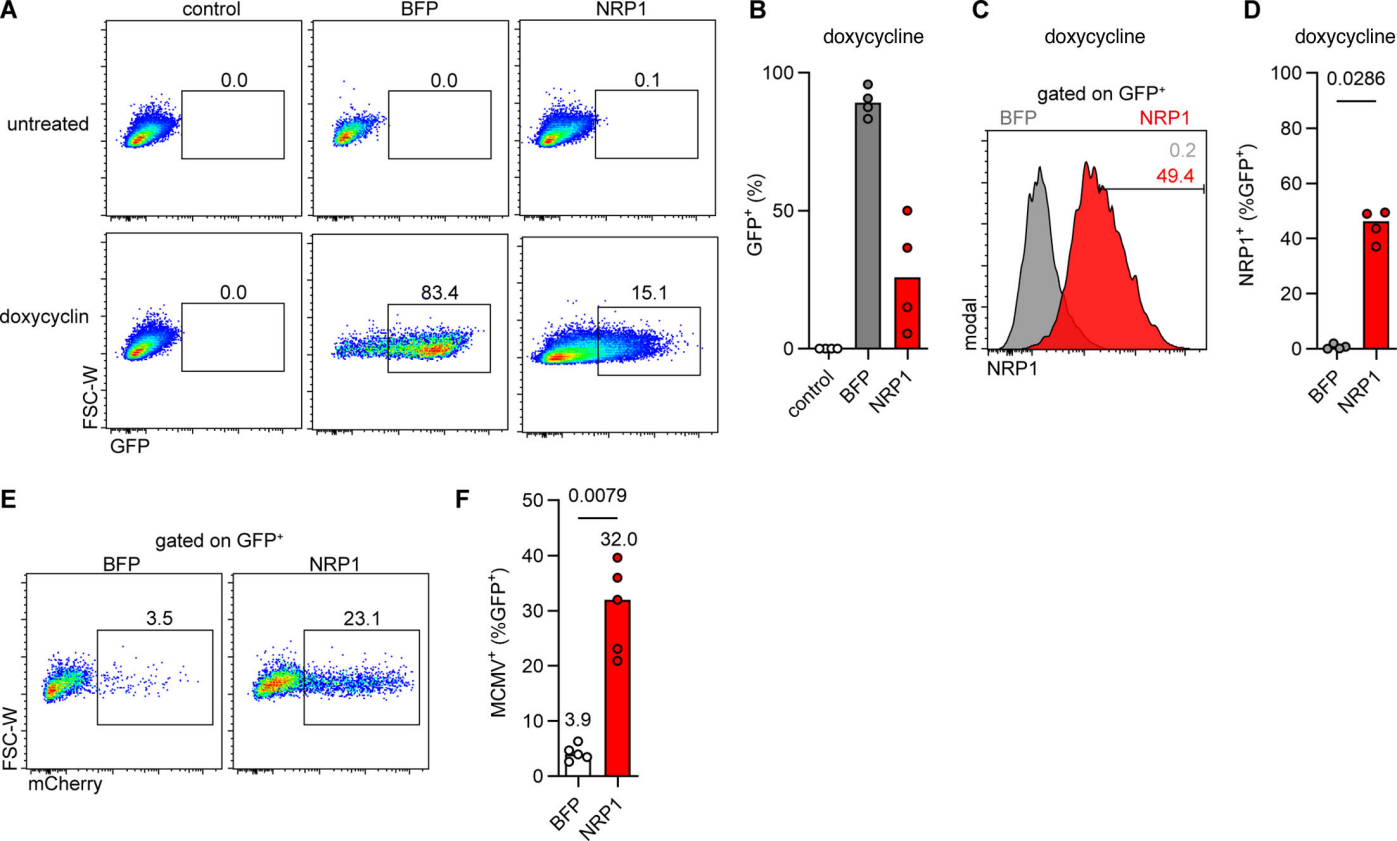
Doxycycline-induced NRP1 promotes MCMV infection in mTSCs. Inducible expression of NRP1 in mTSCs was performed using the *piggyBac* transposon system, followed by analysis for MCMV susceptibility. (**A**) Flow cytometry dot plots and (**B**) percentage of GFP expression in non-transfected, BFP-, or NRP1-transfected mTSCs at 48 h after induction with doxycycline. Representative flow cytometry dot plots and medians of *n* = 2 biological replicates in *n* = 2 independent experiments. (**C**) Flow cytometry histogram of NRP1 surface expression and (**D**) percentage of NRP1^+^ cells among the GFP^+^ cells at 24 h after induction with doxycycline. Representative flow cytometry histogram and medians of *n* = 2 independent experiments with *n* = 2 biological replicates are shown. (**E and F**) NRP1- or BFP-transfected mTSCs were induced with Doxycycline for 48 hpi before being infected with MCMV (MOI 0.5). (**E**) Flow cytometry dot plots of mCherry^+^ cells and (**F**) percentage of MCMV-infected (mCherry^+^) among the GFP^+^ doxycycline-induced mTSCs transduced with BFP or NRP1 at 1 dpi. Representative flow cytometry dot plots and medians of *n* = 3 independent experiments are shown. All *P*-values depicted were calculated using the Mann-Whitney U test.

NRP1 is involved in vessel sprouting, and global deletion of NRP1 leads to lethality in mice due to impaired yolk sac vascularization (44). Overexpression of this protein may thus negatively impact placenta development. To assess whether transient expression of NRP1 is sufficient to increase MCMV infection in mTSCs, a doxycycline-inducible expression system was used. As several attempts to transduce mTSCs with lentiviral vectors were unsuccessful in our hands, a piggyBac transposon system allowing doxycycline-inducible transgene expression was applied (45). The transgene is followed by an internal ribosome entry site (IRES) and GFP. PiggyBac transposon plasmids encoding NRP1-IRES-GFP or BFP-IRES-GFP were constructed and used for the transfection of mTSCs. After puromycin selection, doxycycline-inducible transgene expression was analyzed by flow cytometry (Fig. 7A). Doxycycline led to GFP expression in cells transfected with either construct (Fig. 7A and B), whereas NRP1 expression was observed in cells transfected with the NRP1-IRES-GFP but not the BFP-IRES-GFP construct (Fig. 7C and D). MCMV infection led to a significant increase in the percentage of mCherry^+^ cells in doxycycline-treated mTSCs expressing NRP1, when compared to the BFP control cells (Fig. 7E and F). These data demonstrated that transposon-mediated genetic modification of placental trophoblast cells allowed increased MCMV infection via NRP1 expression.

## DISCUSSION

Congenital CMV infection causes a significant health burden worldwide (46). Unfortunately, the most widely used and well-established animal model for CMV disease has limited applicability for studying virus-host interactions in the fetus. Although infection of neonatal mice with MCMV models aspects of congenital CMV disease, MCMV cannot be used to study CMV transmission to the fetus *in utero* and congenital CMV infection. In the present study, we addressed tissue-specific features of the murine materno-fetal barrier interfering with vertical MCMV infection. We employed an intravenous high-dose MCMV infection model, which, while limiting the observation period due to high viral loads in organs such as the liver, enabled direct exposure of cell-free virus to the materno-fetal interface. In immunocompromised mice, MCMV replicated to considerably higher virus loads in the livers than in WT mice. However, even under these conditions, MCMV-infected cells were hardly detected in cells of the materno-fetal barrier. In line, *ex vivo* exposure of primary placental cells, SM9-1 trophoblasts, and mTSCs to MCMV revealed a cell type-specific resistance to infection. NRP1 expression reversed this phenotype and increased MCMV susceptibility.

NRP1 was recently identified as a critical host factor for MCMV, and genetic deletion of NRP1 in SVEC4-10 cells abrogated MCMV infection and gene expression (25). MHC-I expression was described to be required for MCMV infection of macrophages (24), and we found β2-M, as part of the MHC-I, was very low in SM9-1 cells and mTSCs. How enforced MHC-I expression impacts infectibility in these cells remains to be tested in future studies. We found NRP1 to be expressed in MCMV-susceptible cell lines but not in resistant SM9-1 cells and mTSCs. Expression of NRP1 abrogated the resistance toward MCMV infection in these placental cells. Interestingly, transfection or transduction of SM9-1 cells or mTSCs with NRP1 led to a lower frequency of reporter fluorophore expression than the respective controls. This may result from the detrimental effects of NRP1 expression in trophoblasts and warrants further investigation. NRP1 is one of two homologous transmembrane proteins with a short cytoplasmic domain and a large extracellular region, which is divided into three domains. The extracellular domain allows binding to several ligands, including members of the semaphorin and vascular endothelial growth factor families, and thus NRP1 can form complexes with other transmembrane receptors to act as a co-receptor in various biological processes (40). NRP1 has been reported to act as an entry receptor for human T-cell lymphotropic virus type 1 (47, 48), Epstein-Barr virus (49), Kaposi’s sarcoma-associated herpesvirus (50), and the severe acute respiratory syndrome coronavirus 2 (51, 52). The related NRP2 has been identified as a host factor for Lujo virus infection (53). Importantly, NRP2 is also a receptor for HCMV, allowing virus entry into epithelial and endothelial cells (22). The HCMV pentameric envelope glycoprotein complex, which is necessary for viral infection of several cell types, including epithelial cells, has been identified as the interaction partner for NRP2 (22, 54), and more recently, NRP2 has been suggested to facilitate guinea pig CMV infection (55). It seems likely that NRP1 serves as a receptor for MCMV entry into specific cells, but this has not been formally demonstrated yet. Hence, NRP1 has merely been called a host factor required for infection of particular cells (25). Interestingly, the MCMV-encoded chemokine 2 (MCK2), a part of the gH-gL-MCK2 glycoprotein complex that is analogous to the HCMV pentameric complex (56), was reported not to be involved in NRP1-dependent MCMV infection (24, 25). Further studies will be necessary to determine how NRP1 facilitates MCMV infection, whether it acts as an entry receptor, and which viral glycoproteins bind to it.

CMVs are known to cross the placenta in humans, non-human primates, and guinea pigs but not in rodents such as mice or rats (13). The microanatomy of the materno-fetal interface is composed of various cell layers with maternal and fetal origin and is substantially different in the aforementioned species. Humans, non-human primates, and guinea pigs are hemomonochorial, whereas mice are hemotrichorial, meaning that one versus three trophoblast layers separate the chorion from maternal blood, respectively (57, 58). There are several possibilities for MCMV vertical transmission to the fetus such as (i) infection of trophoblast cells and further infection of fetal cells via cell-to-cell spread, (ii) cell-free virus or infected maternal cells passing through leaks of the materno-fetal barrier, (iii) virus penetrating the intact syncytiotrophoblast by transcytosis, and (iv) virus-infected maternal cells penetrating the barrier by transmigration. Accordingly, together with fetal vascular endothelial cells, a virus would need to infect or permeate only two cell layers in humans, non-human primates, and guinea pigs to penetrate the materno-fetal barrier, but four cell layers in mice. In addition, CMV is a slowly replicating virus, indicating that the production of virus particles for tissue penetration may require a certain amount of time to allow infection of the placenta and transmission from mother to fetus. Indeed, it has been reported that not until 10 days after intraperitoneal MCMV application, there is evidence of virus replication in placental tissue (7). In this respect, a major difference between species where vertical CMV transmission has been observed versus mice is the comparatively short gestational time of approximately 3 weeks in mice versus ~40, ~23, and ~10 weeks in humans, rhesus macaques, and guinea pigs, respectively (59, 60). Thus, although not addressed in the present study, the combination of anatomical differences together with a short gestation period presumably reduces the chance of vertical MCMV transmission.

In the murine placenta, trophoblasts provide a multilayered defense against viral infection, combining structural, receptor-level, and immune mechanisms. The hemotrichorial murine placenta could reduce cell-to-cell spread in the materno-fetal interface. Resistance is further supported by restricting the expression of viral entry receptors. While human and guinea pig trophoblasts exhibit expression of NRP2 (55, 61), murine trophoblasts display low levels of NRP1. The role of adaptive immune response was observed by MCMV infection of pregnant SCID mice bearing the *Prkdc^scid^* mutation, where increased infection in E18 fetal membranes was observed (10). Likewise, we found a few placental cells to be infected in *Rag2*^-/-^*Il2rg*^-/-^ mice, which lack T and NK cells (62, 63). Thus, lymphocytes likely interfere with materno-fetal MCMV infection. When viruses bypass receptor-level restriction, murine trophoblasts themselves mount innate immune responses. Trophoblasts can sense and respond to their microenvironment through pattern recognition receptors, and placenta-derived type I interferon, together with their downstream interferon-stimulated genes, contributes to protecting against viral infection (64). The role of type I interferons for placental antiviral defense is supported by the observation that pregnant *Ifnar1*^⁻/⁻^ mice exhibited Zika virus infection in trophoblasts (65). Further studies are needed to decipher the role of adaptive and innate immune responses and what the underlying mechanisms are.

The present study suggests that the absence of NRP1 expression in murine placental trophoblast cells confers resistance against MCMV infection, reducing tissue damage in the materno-fetal barrier and potentially decreasing the risk of vertical transmission to the fetus.

## MATERIALS AND METHODS

### Animals

All mice were on a C57BL/6 background and kept in individually ventilated cages under specific pathogen-free conditions according to the recommendations of the FELASA (66). *Pasteurella pneumotropica*, *Helicobacter species*, and *murine norovirus 1* were detected in sentinel animals tested in this breeding barrier. Food and water were provided *ad libitum. Rag2*^-/-^*Il2rg*^-/-^ (B6(Cg)-Rag2^tm1.1Cgn^/J—JAX008449 crossed with B6.129S4-Il2rg^2tm1Wjl^/J—JAX003174), *Ifnar1*^-/-^ (B6(Cg)-Ifnar1^tm1.2Ees^/J—JAX028288), and actb-EGFP (C57BL/6-Tg(CAG-EGFP)131Osb/LeySopJ-JAX006567) were bred locally, C57BL/6J *WT* mice (JAX000664) were purchased from Charles River Laboratories (Sulzfeld).

### Cell lines

SM9-1 cells, an immortalized trophoblast cell line, were a kind gift from Joan Hunt (University of Kansas Medical Center, USA) and Margaret Petroff (Michigan State University, USA). They originated from a gestational day 9 Swiss-Webster mouse placenta, and the non-adherent trophoblastic cell outgrowths from the placental explants were transferred for 5–6 passages. These cells are immortalized but not transformed. In this study, SM9-1 cells were grown in RPMI 1640 containing antibiotics and supplemented with 10% fetal calf serum (FCS) and 0.05 mM β-mercaptoethanol at 37°C, 5% CO2 as previously described (34). All cell lines were cultured under identical conditions to allow comparability.

mTSCs were isolated and cultured as previously described (43). Briefly, the cells were kept undifferentiated in 30 vol% TS medium (RPMI 1640 containing 20% FCS, 100 IU/mL penicillin, 100 mg/mL streptomycin, 1 mM sodium pyruvate, and 0.1 mM β-mercaptoethanol) and 70 vol % MEF-conditioned medium (TS medium harvested from mitomycin C-treated primary MEFs) supplemented with 30 ng/mL FGF4 and 1.2 μg/mL heparin. To induce differentiation, mTSC cells were cultured in TS medium only.

### Statistical analysis

We performed statistical tests as indicated and provided levels of significance directly for each analysis of interest. Data were processed using Prism software (GraphPad).

### MCMV infection

The MCMV-3DR recombinant, which was used in all experiments, was generated from the pSM3fr bacterial artificial chromosome by BAC recombineering. It encodes *Gaussia* luciferase, mCherry, contains a sequence within the *m164* ORF encoding the SIINFEKL peptide, and contains the complete *Mck2* ORF, but its *m157* ORF is replaced by the sequences for the reporter proteins (31, 67, 68). Virus stocks were produced on 10.1 immortalized mouse embryonic fibroblasts (69), purified by centrifugation through a sucrose cushion, and titrated on M2-10B4 mouse bone marrow stromal cells (ATCC CRL-1972). To ensure consistent infectious unit concentrations in virus stocks, plaque assays were routinely performed using both the previous and newly prepared stocks in parallel. For *in vivo* infection experiments, animals were anesthetized (isoflurane) and received a single intravenous injection of 10^6^ PFU MCMV-3DR.

### Histology and data processing

Organs were fixed in PBS-buffered 2% paraformaldehyde with 30% sucrose. Organ slices (7 µm thick) were prepared and stained with DAPI. Images were acquired with an AxioScan Slide Scanner (Carl Zeiss) using the Colibri 7 LED light source and processed as TIFF-formatted files with ZEN (Carl Zeiss) and ImageJ (NIH) software. Single-channel exports were converted into binary images, and particles were counted as nuclei (50–200 µm^2^ area for DAPI^+^ signals) or MCMV-infected cells (90–600 µm^2^ mCherry^+^ signals), applying the “classic watershed” plugin. Ratios of mCherry^+^ to DAPI^+^ signals were determined for each image.

### Luciferase assay

Cell culture supernatants were measured for luciferase expression by quantification of luminescence after the addition of native coelenterazine (Synchem) with a Centro XS³ LB 960 luminometer (Berthold Technologies) essentially as described previously (15).

### Primary cell isolation

Single-cell suspensions were prepared from the placenta at gestational day 12.5. Placenta cells were generated as described in references (70, 71). Briefly, after macroscopic dissociation from the fetus, placenta fragments were digested in collagenase type II-S (Sigma-Aldrich) and then passed through a 70 µm cell strainer before further separation via a Percoll (Sigma-Aldrich) gradient and subsequent resuspension in DMEM/F12 medium supplemented with 10% FCS, 100 IU/mL Penicillin, 100 µg/mL Streptomycin, 1 mM sodium pyruvate, and 0.05 mM β-mercaptoethanol.

### Quantitative real-time RT-PCR

Total RNA was extracted from the cells using the innuPREP RNA Mini Kit (Analytik Jena), and contaminating DNA was removed using the TURBO DNA-free Kit (Ambion). cDNA was synthesized from 1 to 5 µg of the extracted RNA by using the RevertAid H Minus Reverse Transcriptase, oligo-dT primers, and the RNase inhibitor RiboLock (Thermo Fisher Scientific). qPCR was performed on a QuantStudio 3 (ThermoFisher Scientific) and the PowerTrack SYBR Green Mastermix (Fisher Scientific). To amplify mouse transcripts, the following primers were used: *Gapdh* (GGAGAAACCTGCCAAGTATGATG and GACAACCTGGTCCTCAGTGTAGC), *Cdx2* (AGACAAATACCGGGTGGTGTA and CCAGCTCACTTTTCCTCCTGA), *Pl1* (GACTACCCTGCTTGGTCTGG and GAAAGACAACTCGGCACCTC), and *Tpbpa* (CAGAGAGTGGCGATGGGTTTT and GACAATGGCACAGTGGCTGTT). Transcript levels were normalized to a housekeeping gene (*Gapdh*).

### Immunoblot analysis

For immunoblot analysis, cells were lysed in NP-40 buffer (50 mM Tris, 150 mM NaCl, 1% Nonidet P-40, and Complete Mini protease inhibitor cocktail [Roche]), separated by SDS-PAGE, and subsequently transferred to a nitrocellulose membrane by semi-dry blotting. Target proteins were detected using monoclonal antibodies against NRP1 (D62C6, Cell Signaling Technology) or GAPDH (14C10, Cell Signaling Technology) and combined with HRP-coupled secondary antibodies (Jackson ImmunoResearch). Recombinant mouse NRP1 (R&D Systems) was included as a positive control. Images were acquired with the Fusion Capture Advance FX7 16.15 (Peqlab) camera.

### Transfection

SM9-1 trophoblast cells were transfected with expression plasmids pEGFP-C1 (Clontech) or pCMV3-NRP1-GFPSpark (SinoBiological) plasmids using Lipofectamine 2000 (Thermo Fisher Scientific). Briefly, 3 µg of plasmid was mixed with 10 µL of Lipofectamine 2000 in Opti-MEM (GIBCO) for 10 min at room temperature before being added dropwise to 3 × 10^5^ SM9-1 cells seeded the day before in a six-well plate.

mTSCs were transfected with expression plasmids pEGFP-C1 and pCMV3-NRP1-GFPSpark using GenJet transfection reagent (SignaGen). Briefly, 1 µg of plasmid was mixed with 3 µL of GenJet in DMEM for 15 min at RT before being added dropwise to 1 × 10^5^ mTSCs seeded the day before in a 12-well plate.

### Transduction with lentiviral expression vectors

NRP1-GFPSpark or GFPSpark coding sequences were PCR-amplified from the pCMV3-NRP1-GFPSpark plasmid using forward (TATAGAATTCATGGAGAGGGGGCTGCCG or TATAGAATTCATGGTGAGCAAGGGCGAGGAGC) and reverse (TTAAGAATTC TTACTTGTACAGCTCGTCCATGCCG) primers containing EcoRI restriction sites. The PCR product was cleaved with EcoRI and inserted into the lentiviral vector pLeGO-iB2-puro (72). This vector contains an SFFV promoter driving the expression of the insert, followed by IRES2-BFP-P2A-puromycin. Lentiviruses were generated using standard third-generation packaging vectors in HEK-293T cells. SM9-1 cells were transduced with the lentiviral vectors expressing NRP1-GFPSpark or GFPSpark in the presence of polybrene (Sigma). Transduced SM9-1 cells were selected with 2.5 µg/mL puromycin (Sigma), and GFP-positive cells were sorted using FACS Aria Fusion.

### *PiggyBac* transposon system

NRP1- and BFP-coding sequences were PCR amplified from the pCMV3-NRP1-GFPSpark plasmid or from the pLEGO-iB2 plasmid using forward (ATGGAGAGGGGGCTGCCG or ATGAGCGAGCTGATTAAGG) and reverse (TCACGCCTCTGAGTAATTACTCTG or TTACTTGTACATCAGGCGC) primers and inserted into the Tet-on *piggyBac* vector (Addgene # 97421) modified as described in reference (73). The vector contains a doxycycline-inducible TREG3 promoter driving the expression of the insert, followed by IRES-GFP, while the EF-1α promoter drives the expression of the reverse tetracycline-controlled transactivator and puromycin resistance marker. 2 × 10^5^ mTSCs were seeded in a six-well plate the day before transfection. mTSCs were transfected with 2 µg PB-NRP1 or PB-BFP and 1 µg of pCMV-HyPBase (Sanger Plasmid Repository) encoding a hyperactive BP transposase (74) using 6 µL GenJet transfection reagent. 72 h post-transfection, cells were selected with 0.5 µg/mL of puromycin. NRP1 or BFP and GFP expression was induced by adding 2 µg/mL doxycycline (Biomol).

### Viral replication kinetics

SM9-1 or mTSC cells (3 × 10^4^) were infected at an MOI of 1 with MCMV-3DR in triplicates. The input virus was removed 4 h later, and fresh medium was added. The supernatants were harvested at different times post-infection and titrated by plaque assay on M2-10B4 cells.

### Flow cytometry

Cells were acquired on a LSRFortessa Cell Analyzer or FACSymphony A1 Cell Analyzer (BD Biosciences). The following antibodies were used for stainings: CD45-APC (30-F11), NRP1-PE (3E12), PDGFRα-PerCP-Cy5.5 (APA5), β2-microglobulin-APC (A16041A), integrin β1-PE-Cy7 (HMβ1-1), and NRP1-APC (3E12). Data were processed with FlowJo version 10 (BD Biosciences). Cells were pre-gated in a forward to sideward scatter plot, and cell duplicates were excluded in a second forward scatter high to area plot. Dead cells were identified by the use of a cell viability marker (Zombie Violet or NIR) and excluded from analysis.

### Reanalysis of a single-nuclei RNA-sequencing data set

A single-nuclei RNA sequencing data set (NCBI GEO [GSE152248]) (41) was reanalyzed using the R package Seurat (v 4.3.0.1). The cell subsets were annotated according to the original report. For analysis, only placentas isolated at gestational day 12.5 were included.

## Data Availability

All data supporting the findings of this study are available within the article.

## SUPPLEMENTAL MATERIAL

Supplemental figures jvi.01610-25-s0001.docx Figures S1 and S2.

ASM does not own the copyrights to Supplemental Material that may be linked to, or accessed through, an article. The authors have granted ASM a non-exclusive, world-wide license to publish the Supplemental Material files. Please contact the corresponding author directly for reuse.
