# Supplementary material for: Neuropilin-1 expression modulates infection susceptibility to murine cytomegalovirus at the materno-fetal interface
Source: J Virol. 2025 Nov 25;99(12):e01610-25. doi: 10.1128/jvi.01610-25 (PMC12724362; doi:10.1128/jvi.01610-25)
Supplement: Supplemental figures — Figures S1 and S2. [file jvi.01610-25-s0001.docx]

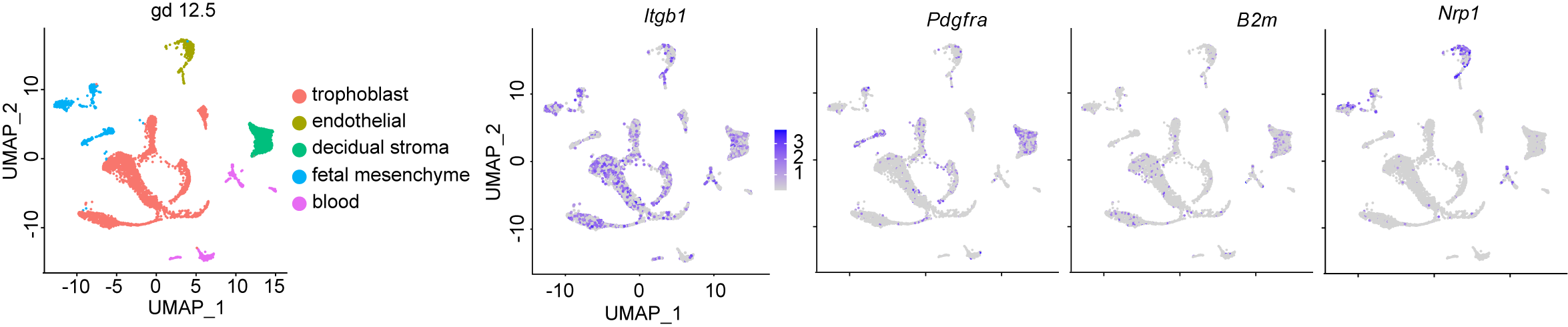


**Supplemental Figure 1. scRNA expression in gd 12.5 mouse placental cells**

Visualization of placenta nuclei transcriptome similarity as described in (41) using uniform manifold approximation and projection (UMAP) including (left) the annotation as described and (right) the expression of putative MCMV entry receptor genes as indicated.


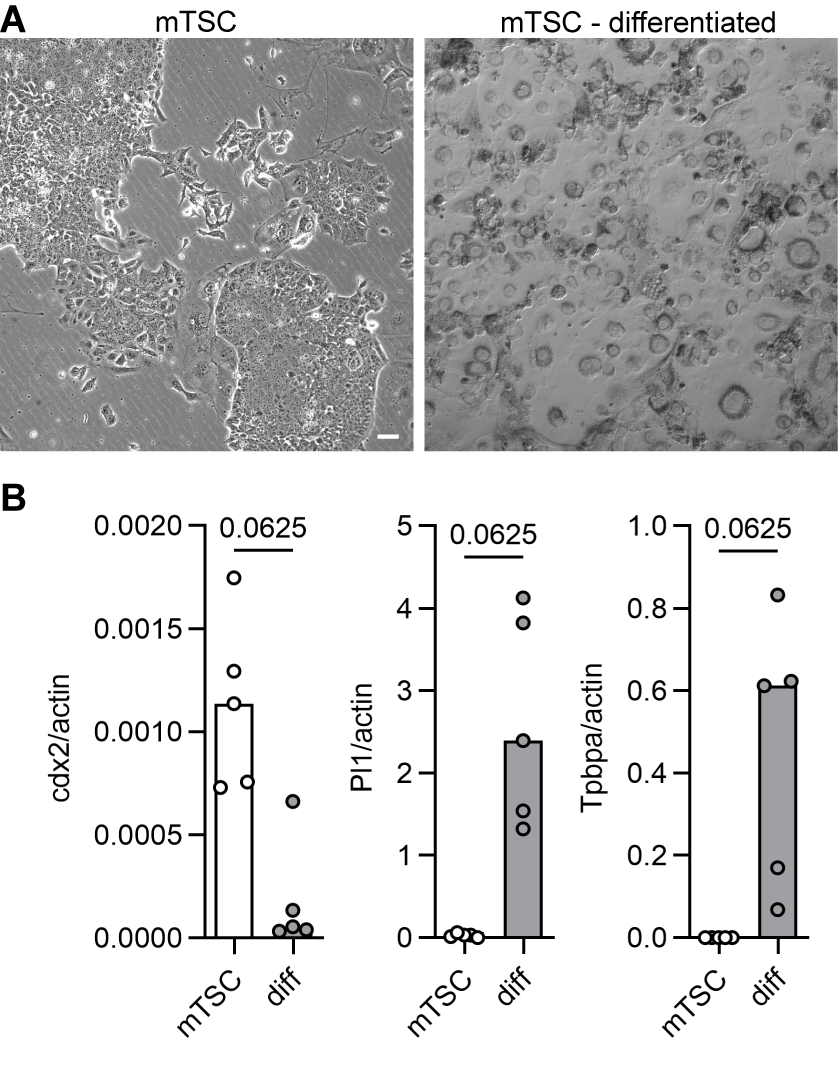


**Supplemental Figure 2. Differentiation of mTSC**

**A)** Brightfield microscope images of mTSC (left) and differentiated cells eight days after medium change (right); scale bar: 100 µm.

**B)** RNA was extracted from mTSCs or differentiated cells (8 days) and analyzed for the lineage marker genes as indicated by quantitative RT-PCR. Median of five independent experiments is shown. P-values were calculated using the Wilcoxon matched-pairs signed rank test.
